# Evolution of Health-Related Quality of Life Among Migrant Minors in Transit to the United States According to their Socioeconomic Context: An Exploratory Cross-Sectional Study in Mexico

**DOI:** 10.1007/s10903-026-01941-9

**Published:** 2026-06-16

**Authors:** Zeus Aranda, Ana-Cristina Sedas, Daniel Bernal-Serrano, José Pulido-Manzanero, Enrique Regidor, Anna M. Mandalakas, Karla Fredricks

**Affiliations:** 1 Departamento de Salud Pública y Materno-Infantil, Facultad de Medicina, Complutense University of Madrid, Madrid, Spain; 2 Division of Global Health, Department of Pediatrics, Baylor College of Medicine, Houston, USA; 3 Center for Humanitarian Health, Bloomberg School of Public Health, Johns Hopkins University, Baltimore, USA; 4 Escuela de Gobierno y Transformación Pública, Monterrey Institute of Technology and Higher Education, Mexico City, Mexico; 5 Centro de Investigación Biomédica en Red de Epidemiología y Salud Pública, Madrid, Spain

**Keywords:** Child health, Adolescent health, Migrant health, Refugee health, Social determinants of health, Mexico

## Abstract

The population of migrant and refugee children and adolescents increased in the first two decades of the 21st century in the Americas region. This analysis was conducted with the aim of shedding light on the association between the living conditions of migrant minors in Mexico, one of the main transit countries for migrants in the region, and the evolution of health-related quality of life throughout the migration process. Data were collected on 192 minors aged one year or older in transit to the United States through their caregivers in shelters on Mexico’s northern border, including sociodemographic/socioeconomic information and health-related quality of life information prior to departure and during the period of travel. Differences in the evolution of health-related quality of life between origin and travel were explored for different categories of sociodemographic/socioeconomic variables. Several social groups experienced a greater deterioration in some aspects of health-related quality of life, such as those in more precarious situations at origin and along the route, younger minors, or minors whose primary caregivers identified as Afro-descendants and/or indigenous. Minors who had experienced child abuse in their place of origin and adolescents experienced improvements in certain aspects of their health-related quality of life throughout the journey. Despite the exploratory nature of the analysis, it constitutes a first step in identifying populations of children and adolescents on the move in Mexico who should be prioritized for interventions aimed at promoting their well-being.

**Supplementary Information:**

The online version contains supplementary material available at 10.1007/s10903-026-01941-9.

## Introduction

In the first two decades of the 21st century, there was a large increase in the mobile population in the Latin America and Caribbean (LAC) region (from 24.6 to 42.9 million international migrants) [1]. This mobility has been accompanied by an increase in the proportion of women and children, who migrate for various reasons, including organized crime violence, political repression, or economic problems [2]. One of the countries with the highest flow of migrants is Mexico, which, due to its northern border with the United States (US), is one of the main transit countries for people on the move in the Americas.

For a large part of the mobile population, precarious living conditions in their country of origin persist and even worsen throughout the migration journey, with added vulnerabilities such as lack of regular migration status, lack of knowledge of protection services in transit countries, racist or xenophobic discrimination, lack of stable income, and unhealthy living conditions [2]. Children are particularly vulnerable due to their early stage of development, which exacerbates the impact of these living conditions on their well-being. However, despite the high levels of vulnerability and growing demographic relevance of children on the move in Mexico, there are still very few studies on the health status and living conditions of this population. This analysis sheds light on the relationship between the sociodemographic/socioeconomic characteristics of migrant minors in their place of origin and during their journey, and the evolution of their health-related quality of life throughout the migratory journey.

## Methods

### Study Design, Setting, and Participants

This is a cross-sectional observational study. Data collection was carried out in 12 different shelters in Piedras Negras, Saltillo, and Monterrey, Mexico. Surveys were conducted with 118 adult migrant primary caregivers in transit to the US about the minors in their care, selected using non-probability sampling for convenience. The study used United Nations Children’s Fund (UNICEF)’s definition of primary caregivers, understood as the adults responsible for performing the main caregiving duties for the child in daily life [3]. Among the study participants, most primary caregivers were the minor’s parents (more than 96%), with a minority of cases involving a grandmother or an uncle. Surveys were conducted with all caregivers of minors identified during visits to participating shelters until information was obtained on 200 minors, which is consistent with the sample size used in the International Organization for Migration Displacement Tracking Matrix (IOM DTM) surveys in Mexico and in similar studies of migrant populations in transit that employ non-probability sampling methods [4]. Speaking Spanish was not a requirement for participation in the study; however, it was the primary language in all cases except for one Guatemalan caregiver, for whom the primary language was Ixil. Despite this, this participant considered herself a native Spanish speaker and had no difficulty communicating with the research team in Spanish. None of the caregivers identified declined to participate in the survey, and the participants received no compensation for taking part in the study. Data collection was conducted between July and September 2024. For the present analysis, only data on minors aged between one and 17 years (*n* = 192) were used.

### Data Collection

The surveys included questions about the sociodemographic/socioeconomic characteristics of the caregivers and the minors in their care (living conditions for the last year prior to departure and throughout the migration journey), as well as health-related quality of life questionnaires: the Infant Toddler Quality of Life Questionnaire (ITQOL) for minors aged 1 to 4 (47 questions) and the Child Health Questionnaire (CHQ) for minors aged 5 to 17 (28 questions) (QualityMetric, 2024). The ITQOL and CHQ questionnaires were answered retrospectively for the month prior to departure and the last month of the migratory journey. The questionnaire was read in Spanish to the participants by the first author, who recorded the responses in a CommCare app (Dimagi, 2024).

### Data Analysis

The values for four subscales (equivalent in ITQOL and CHQ to combine data for minors aged 1–4 and 5–17) were selected as health-related quality of life variables: bodily pain/discomfort, global behavior (refers to an assessment of the minor’s overall behavior in comparison with other minors of the same age), general perception of health, and family cohesion. Scores ranging from 0 to 100—with 100 being the best—were calculated using the PRO CoRE tool (QualityMetric, 2024). Next, the average value of the four quality of life subscales was calculated for the last month before departure, the last month during the migratory journey, and the difference between the two periods, as well as their corresponding standard deviations for the different categories of sociodemographic/socioeconomic variables. Student’s t-tests (two categories) or ANOVA (three categories) were performed to compare the differences in subscale scores between origin and travel between the different categories. Tests with a *p*-value < 0.05 were considered statistically significant. The analysis was performed using STATABE 18 (StataCorp LLC, 2024).

## Results

### Bodily Pain/Discomfort Subscale

A greater deterioration in bodily pain/discomfort was identified throughout the migration process in minors with unmet health needs or difficulties in paying for utility bills in their place of origin, as well as in those who did not sleep indoors, did not have access to toilets, did not have enough drinking water, did not have enough nutritious food, did not have access to hygiene measures, or did not have enough clothing during half or most of the journey (Table 1 and Supplementary Table 1).

**Table 1:** Comparison of the evolution of different aspects of health-related quality of life among migrant minors with different sociodemographic/socioeconomic profiles (*N* = 192)

Subscales and variables*	Categories (*n*)	Average subscale score last month prior to departure (SD)	Average subscale score last month of travel (SD)	Average subscale score difference (travel-departure) (SD)	*p*-value¹
*Bodily pain/discomfort subscale*					
*Socioeconomic variables at origin*					
Always received care when in need of health services during the year prior to departure	Yes (147)	94.39 (17.07)	80.8 (23.93)	-13.59 (27.35)	0.0021
	No (39)	97.69 (10.12)	68.33 (31.3)	-29.36 (30.83)	
Difficulty paying for utility bills	Yes (19)	95.79 (12.61)	63.29 (33.91)	-32.5 (31.83)	0.0099
	No (173)	95.17 (16)	80.35 (24.43)	-14.83 (27.63)	
*Socioeconomic variables at the journey*					
Sleeping indoors	Half of the days or less (107)	97.57 (10.08)	75.07 (26.68)	-22.5 (26.15)	0.0011
	Most of the days (85)	92.29 (20.36)	83.18 (24.33)	-9.12 (29.67)	
Access to toilets	Half of the days or less (109)	96.7 (13.13)	74.47 (26.62)	-22.22 (27.29)	0.0015
	Most of the days (83)	93.31 (18.4)	84.16 (24.03)	-9.16 (28.46)	
Enough drinking water	Half of the days or less (105)	96.71 (13.93)	74.74 (25.92)	-21.98 (27.5)	0.0036
	Most of the days (87)	93.45 (17.46)	83.39 (25.26)	-10.06 (28.43)	
Enough nutritious food	Half of the days or less (139)	95.68 (14.81)	74.66 (26.23)	-21.03 (27.38)	0.0004
	Most of the days (53)	94.06 (17.84)	89.15 (22.07)	-4.91 (28.26)	
Access to hygiene measures/products	Half of the days or less (144)	95.87 (14.9)	76.23 (26.02)	-19.64 (27.99)	0.0096
	Most of the days (48)	93.33 (17.82)	85.94 (24.44)	-7.4 (28.25)	
Sufficient clothing, appropriate for the weather	Half of the days or less (132)	96.25 (13.63)	75.78 (25.92)	-20.47 (27.28)	0.0046
	Most of the days (60)	93 (19.53)	85 (24.97)	-8 (29.4)	
*General health perception subscale*					
*General sociodemographic variables*					
Age (years)	1–4 (51)	75.42 (28.88)	70.6 (27.56)	-4.82 (14.2)	0.0367
	5–11 (102)	78.57 (27.63)	82.88 (18.15)	4.31 (22.31)	
	12–17 (39)	81.19 (25.72)	80.64 (25.78)	-0.54 (24.18)	
*Socioeconomic variables at origin*					
Child abuse	Yes (58)	68.53 (33.54)	74.06 (25.64)	5.53 (-1.32)	0.0458
	No (134)	82.48 (23.38)	81.37 (21.61)	-1.1 (18.4)	
*Family cohesion subscale*					
*General sociodemographic variables*					
Age (years)	1–4 (51)	75.1 (23.1)	66.57 (28.36)	-8.53 (26.89)	0.019
	5–11 (102)	73.92 (24.85)	65.88 (25.55)	-8.04 (21.92)	
	12–17 (39)	64.23 (23.41)	67.56 (23.53)	3.33 (17.82)	
Indigenous/Afro-descendant identity of caregiver	Yes (15)	69.33 (28.28)	52 (24.19)	-17.33 (24.12)	0.0437
	No (177)	72.51 (24.04)	67.63 (25.64)	-4.89 (22.69)	

*Only variables with *p* < 0.05 are shown for the sake of clarity. However, all variables were analyzed for the four subscales under study (bodily pain/discomfort, general health perception, global behavior, and family cohesion).

¹Comparison of the average difference in scores (travel-departure) for different categories of sociodemographic/socioeconomic variables using Student’s t-tests or ANOVA.

### Global Behavior Subscale

No significant association was observed between the sociodemographic/socioeconomic variables studied and changes in the global behavior of minors (Supplementary Table 2).

### General Health Perception Subscale

A significant association was identified between the age of the minors and the evolution of general health as perceived by caregivers, with the 1–4 age group showing a decrease of 4.82 points on the subscale, the 5–11 age group showing an increase of 4.31 points, and the 12–17 age group showing a decrease of 0.54 points. There was also a significant difference between the change in the general health of minors who suffered abuse at home (5.53), which improved, compared to those who did not (-1.1) (Table 1 and Supplementary Table 3).

### Family Cohesion Subscale

Associations were identified between the age of the minors and the caregiver’s ethnic identity and changes in the level of family cohesion between origin and journey (Table 1 and Supplementary Table 4). A decrease was observed in the family cohesion subscale in minors aged 1–4 (-8.53) and 5–11 (-8.04), in contrast to an improvement in minors aged 12–17 (3.33). Minors whose caregiver identified as Afro-descendant or indigenous showed greater deterioration in family cohesion than minors not belonging to this group.

## Discussion

Our study found associations between the inability to meet the health needs of minors and difficulty paying utility bills for services in the place of origin, as well as deficiencies in housing, WASH (water, sanitation, and hygiene), nutrition, and clothing during the journey and the change of the level of bodily pain/discomfort throughout the journey, while none of these factors were related to changes in the global behavior of minors. Age and having suffered abuse in the place of origin were associated with changes in the perceived general health of minors throughout the journey, while the age of the minor and the ethnic identity of the caregiver were associated with changes in family cohesion.

Difficulties in paying for utility bills, as well as the inability to meet the health needs of minors, may be indicators of the economic difficulties experienced by many migrant families in their country of origin, which continue or worsen throughout the journey [5]. The precariousness of this population is reflected in the multiple deficiencies experienced throughout the journey, associated with increased bodily pain/discomfort. It is important to note that, due to the tightening of US immigration policies since this study was conducted, the decline in migrant minors’ well-being associated with harsh living conditions during the migration journey is likely even greater today. A significant portion of the migrant population traveling to the US from Mexico remains stranded in border cities, exposed to precarious living conditions for months [6]. Furthermore, many of the initiatives that provide shelter, food, drinking water, clothing, and sanitation facilities to the migrant population throughout LAC—most of which are run by civil society organizations—have seen a significant reduction in their resources due to funding cuts from multilateral and bilateral agencies over the past year [7]. Our findings underscore the importance of maintaining the resources needed to ensure decent living conditions for migrant children and their families to prevent the deterioration of their health throughout the migration process.

Differences in perceived general health according to age may be linked to the varying degrees of vulnerability faced by minors depending on their level of development, with minors under five being more vulnerable to infectious diseases (considering also the discontinuation of immunizations), nutritional problems, or greater difficulty adapting to a constantly changing environment [8]. On the other hand, the improvement in the perceived general health of minors who suffered abuse in their country of origin may be related to their removal from the people and/or places where the abuse took place, reducing its negative impact on their mental and physical health throughout the journey [9].

Regarding family cohesion, the migration situation can exacerbate existing relationship problems or create new ones, due to the challenges arising from the migration journey, such as the reduction or disappearance of the childcare support network, which can have a particular impact on families with young children [10]. However, as shown in the case of adolescents, new circumstances can also represent an opportunity to redefine roles that involve improvements in coexistence [11]. Caregivers of African descent or indigenous origin reported greater deterioration in family cohesion than caregivers who did not belong to these ethnic groups, which may be related to the greater discrimination experienced by racialized people and its impact on intra-family relationships [12]. The implementation of parenting interventions aimed at caregivers in mobility could be an effective strategy for improving intra-family relationships and ultimately the well-being of caregivers and minors [13]. Parenting programs for migrants previously implemented by coalitions of civil society and academia along Mexico’s northern border could be scaled up, such as *Mamá Empoderada* [14] and *Crianza con Amor* [15].

### Limitations

Although information on quality of life was collected for two periods, the data in the country of origin were collected retrospectively, with potential recall bias. Another limitation lies in the sample size that participated in the study, which was too small for the development of regression models with multiple predictor variables, as well as the type of sampling, which does not ensure the representativeness of the study population and limits the generalization of the findings.

Given the findings and limitations of our study, as well as Mexico’s evolving migration landscape, further research is needed to help clarify the impact of social determinants of health on the well-being of migrant minors. Such research should incorporate new sociodemographic, socioeconomic, and health-related variables and ideally involve larger samples, employ probabilistic sampling, and include longitudinal follow-up of participants to limit the impact of recall bias.

## Conclusion

Our exploratory study identifies sociodemographic/socioeconomic characteristics of migrant minors in Mexico associated with the evolution of their health-related quality of life between their place of origin and their migratory journey. This represents an important first step in understanding the populations and needs that should be prioritized in interventions aimed at improving the well-being of migrant minors.

## Supplementary Information

Below is the link to the electronic supplementary material.

Supplementary Material 1

## Data Availability

The data underlying this article will be shared on reasonable request to the corresponding author.
