# Supplementary material for: Evolution of Health-Related Quality of Life Among Migrant Minors in Transit to the United States According to their Socioeconomic Context: An Exploratory Cross-Sectional Study in Mexico
Source: J Immigr Minor Health. 2026 Jun 16;28(4):1039–44. doi: 10.1007/s10903-026-01941-9 (PMC13451503; doi:10.1007/s10903-026-01941-9)
Supplement: Supplementary file 1 [file 10903_2026_1941_MOESM1_ESM.pdf]

Supplementary Material:

Evolution of health-related quality of life among migrant minors in transit to the United States according to their socioeconomic context: An exploratory cross-sectional study in Mexico

Supplementary Table 1. Comparison of the evolution of bodily pain/discomfort in migrant minors with different sociodemographic/socioeconomic profiles.

	***N* = 192**	**Bodily pain/discomfort subscale**			
	**Categories (*n*)**	Average score last month prior to departure (SD)	Average score last month of travel (SD)	Average score difference (travel-departure) (SD)	*p*-value[1]
**General sociodemographic variables**					
Sex	Male (101)	95.5 (15.42)	79.23 (25.89)	-16.26 (28.67)	0.873
	Female (91)	94.95 (16.03)	78.02 (26.08)	-16.92 (28.42)	
Age (years)	1-4 (51)	94.61 (17.55)	71.81 (28.16)	-22.79 (33.7)	0.1901
	5-11 (102)	95.29 (15.71)	80.78 (25.32)	-14.51 (27.45)	
	12-17 (39)	95.9 (13.12)	82.05 (23.3)	-13.85 (22.55)	
Indigenous/Afro-descendant identity of caregiver	Yes (15)	100 (0)	74 (31.8)	-26 (31.8)	0.1826
	No (177)	94.83 (16.26)	79.05 (25.43)	-15.78 (28.14)	
**Socioeconomic variables at origin**					

Place of origin[2]	Central America/Mexico (148)	94.09 (17.4)	75.93 (26.49)	-18.16 (30.15)	0.1583
	South America (44)	99.09 (6.03)	87.84 (21.77)	-11.25 (21.41)	
Always received care when in need of health services during the year prior to departure	Yes (147)	94.39 (17.07)	80.8 (23.93)	-13.59 (27.35)	0.0021*
	No (39)	97.69 (10.12)	68.33 (31.3)	-29.36 (30.83)	
Unstable or poor housing conditions	Yes (126)	94.29 (16.96)	76.17 (26.32)	-18.12 (29.6)	0.3019
	No (66)	97.05 (12.8)	83.41 (24.64)	-13.64 (26.19)	
Difficulty obtaining food	Yes (147)	94.49 (16)	78.32 (26.11)	-16.17 (29.33)	0.7247
	No (45)	97.67 (9.98)	79.78 (25.54)	-17.89 (25.77)	
Difficulty paying for utility bills	Yes (19)	95.79 (12.61)	63.29 (33.91)	-32.5 (31.83)	0.0099*
	No (173)	95.17 (16)	80.35 (24.43)	-14.83 (27.63)	
Child abuse	Yes (58)	93.28 (18.86)	76.72 (27.46)	-16.55 (30.06)	0.9939
	No (134)	96.08 (14.07)	79.5 (25.28)	-16.59 (27.89)	
**Socioeconomic variables at the journey**					
Caregiver's sense that minor was safe/protected	Half of the days or less (147)	95.58 (15.7)	78.72 (25.42)	-16.85 (28.84)	0.8075
	Most of the days (45)	94.11 (15.71)	78.44 (27.79)	-15.67 (27.58)	

Sleeping indoors	Half of the days or less (107)	97.57 (10.08)	75.07 (26.68)	-22.5 (26.15)	0.0011*
	Most of the days (85)	92.29 (20.36)	83.18 (24.33)	-9.12 (29.67)	
Access to toilets	Half of the days or less (109)	96.7 (13.13)	74.47 (26.62)	-22.22 (27.29)	0.0015*
	Most of the days (83)	93.31 (18.4)	84.16 (24.03)	-9.16 (28.46)	
Enough drinking water	Half of the days or less (105)	96.71 (13.93)	74.74 (25.92)	-21.98 (27.5)	0.0036*
	Most of the days (87)	93.45 (17.46)	83.39 (25.26)	-10.06 (28.43)	
Enough nutritious food	Half of the days or less (139)	95.68 (14.81)	74.66 (26.23)	-21.03 (27.38)	0.0004*
	Most of the days (53)	94.06 (17.84)	89.15 (22.07)	-4.91 (28.26)	
Access to hygiene measures/products	Half of the days or less (144)	95.87 (14.9)	76.23 (26.02)	-19.64 (27.99)	0.0096*
	Most of the days (48)	93.33 (17.82)	85.94 (24.44)	-7.4 (28.25)	
Sufficient clothing, appropriate for the weather	Half of the days or less (132)	96.25 (13.63)	75.78 (25.92)	-20.47 (27.28)	0.0046*
	Most of the days (60)	93 (19.53)	85 (24.97)	-8 (29.4)	
Always received care when in need of health services	Yes (135)	94.81 (16.78)	77.98 (26.57)	-16.83 (29.2)	0.0708
	No (27)	94.44 (16.25)	66.11 (25.88)	-28.33 (33.77)	
	5 or less (72)	96.46 (12.23)	77.53 (25.83)	-18.92 (25.93)	0.5616

Time since leaving place of origin (months)	More than 5 to 10 (77)	93.44 (18.78)	77.08 (26.39)	-16.36 (29.48)	
	More than 10 (43)	96.4 (14.69)	83.37 (25.25)	-13.02 (30.94)	

[1]Comparison of the average difference in scores (travel-departure) for different categories of sociodemographic/socioeconomic variables using Student's t-tests or ANOVA.

[2]The participants from Central America were from Honduras, Guatemala, and El Salvador. The participants from South America were from Venezuela, Colombia, Ecuador, and Peru.

*$p < 0.05$

Supplementary Table 2. Comparison of the evolution of global behavior in migrant minors with different sociodemographic/socioeconomic profiles.

	***N* = 192**	**Global behavior subscale**			
	**Categories (*n*)**	Average score last month prior to departure (SD)	Average score last month of travel (SD)	Average score difference (travel-departure) (SD)	*p*-value[1]
**General sociodemographic variables**					
Sex	Male (101)	67.92 (26.33)	60.69 (25.05)	-7.23 (23.94)	0.9841
	Female (91)	72.31 (27.29)	65 (26.98)	-7.31 (31.41)	
Age (years)	1-4 (51)	73.73 (25.55)	60 (26.55)	-13.73 (27.42)	0.1354
	5-11 (102)	68.28 (27.44)	62.7 (26.59)	-5.59 (28.45)	
	12-17 (39)	69.62 (26.91)	66.41 (23.84)	-3.21 (24.96)	

Indigenous/Afro-descendant identity of caregiver	Yes (15)	69.33 (24.63)	55 (27.58)	-14.33 (37.74)	0.3038
	No (177)	70.06 (27.05)	63.39 (25.84)	-6.67 (26.69)	
**Socioeconomic variables at origin**					
Place of origin[2]	Central America/Mexico (148)	71.52 (27.28)	63.01 (26.92)	-8.51 (28.69)	0.2526
	South America (44)	64.89 (24.77)	61.82 (22.9)	-3.07 (23.68)	
Always received care when in need of health services during the year prior to departure	Yes (147)	70.85 (25.69)	63.37 (26.32)	-7.48 (25.99)	0.9722
	No (39)	66.79 (30.08)	59.49 (24.94)	-7.31 (34.33)	
Unstable or poor housing conditions	Yes (126)	68.25 (28.53)	59.37 (26.21)	-8.89 (27.39)	0.2622
	No (66)	73.33 (23.01)	69.17 (24.54)	-4.17 (28.1)	
Difficulty obtaining food	Yes (147)	68.23 (27.69)	61.56 (26.35)	-6.67 (29.55)	0.5889
	No (45)	75.78 (23.04)	66.56 (24.72)	-9.22 (20.45)	
Difficulty paying for utility bills	Yes (19)	64.21 (33.68)	46.05 (25.85)	-18.16 (36.26)	0.0704
	No (173)	70.64 (25.98)	64.57 (25.43)	-6.07 (26.41)	
Child abuse	Yes (58)	72.84 (28.67)	63.1 (28.67)	-9.74 (33.74)	0.4159
	No (134)	68.77 (25.97)	62.57 (24.87)	-6.19 (24.63)	

**Socioeconomic variables at the journey**					
Caregiver's sense that minor was safe/protected	Half of the days or less (147)	70.07 (27.45)	63.23 (26.64)	-6.84 (27.51)	0.6988
	Most of the days (45)	69.78 (24.89)	61.11 (24)	-8.67 (28.41)	
Sleeping indoors	Half of the days or less (107)	67.01 (26.86)	60.14 (25.47)	-6.87 (27.2)	0.8243
	Most of the days (85)	73.76 (26.41)	66 (26.44)	-7.76 (28.37)	
Access to toilets	Half of the days or less (109)	67.11 (26.68)	59.17 (25.54)	-7.94 (28.62)	0.7015
	Most of the days (83)	73.8 (26.66)	67.41 (26.02)	-6.39 (26.48)	
Enough drinking water	Half of the days or less (105)	66.33 (27.33)	59.95 (24.94)	-6.38 (28.44)	0.6276
	Most of the days (87)	74.43 (25.63)	66.09 (27)	-8.33 (26.8)	
Enough nutritious food	Half of the days or less (139)	68.35 (27.62)	61.08 (25.28)	-7.27 (29.03)	0.9996
	Most of the days (53)	74.34 (24.26)	67.08 (27.59)	-7.26 (23.93)	
Access to hygiene measures/products	Half of the days or less (144)	66.6 (27.66)	60.56 (25.15)	-6.04 (28.2)	0.2894
	Most of the days (48)	80.21 (21.21)	69.27 (27.66)	-10.94 (25.88)	
Sufficient clothing, appropriate for the weather	Half of the days or less (132)	66.86 (27.77)	61.59 (24.81)	-5.27 (27.71)	0.1375
	Most of the days (60)	76.92 (23.31)	65.25 (28.5)	-11.67 (27.24)	
	Yes (135)	62.41 (34.46)	62.78 (26.65)	0.37 (30.03)	0.0979

Always received care when in need of health services	No (27)	72.44 (24.65)	62.74 (26.19)	-9.7 (28.44)	
Time since leaving place of origin (months)	5 or less (72)	68.96 (25.22)	61.32 (25.13)	-7.64 (21.11)	0.8426
	More than 5 to 10 (77)	73.12 (27.17)	65 (25.92)	-8.12 (29.21)	
	More than 10 (43)	66.16 (28.68)	61.05 (27.85)	-5.12 (34.37)	

[1]Comparison of the average difference in scores (travel-departure) for different categories of sociodemographic/socioeconomic variables using Student's t-tests or ANOVA.

[2]The participants from Central America were from Honduras, Guatemala, and El Salvador. The participants from South America were from Venezuela, Colombia, Ecuador, and Peru.

*$p < 0.05$

Supplementary Table 3. Comparison of changes in the general perception of health among migrant minors with different sociodemographic/socioeconomic profiles.

	***N* = 192**	**General health perception subscale**			
	**Categories (*n*)**	Average score last month prior to departure (SD)	Average score last month of travel (SD)	Average score difference (travel-departure) (SD)	*p*-value[1]
**General sociodemographic variables**					
Sex	Male (101)	74.31 (29.89)	75.06 (24.04)	0.75 (21.71)	0.9203
	Female (91)	82.66 (24.05)	83.72 (21.18)	1.06 (20.66)	
Age (years)	1-4 (51)	75.42 (28.88)	70.6 (27.56)	-4.82 (14.2)	0.0367*

	5-11 (102)	78.57 (27.63)	82.88 (18.15)	4.31 (22.31)	
	12-17 (39)	81.19 (25.72)	80.64 (25.78)	-0.54 (24.18)	
Indigenous/Afro-descendant identity of caregiver	Yes (15)	65.14 (36.54)	63.42 (29.48)	-1.72 (26.26)	0.6183
	No (177)	79.38 (26.46)	80.5 (22.05)	1.12 (20.75)	
**Socioeconomic variables at origin**					
Place of origin[2]	Central America/Mexico (148)	76.87 (28.24)	78.07 (22.47)	1.19 (22.15)	0.7259
	South America (44)	82.95 (24.71)	82.86 (24.94)	-0.09 (17.61)	
Always received care when in need of health services during the year prior to departure	Yes (147)	80.94 (25.27)	81.71 (20.61)	0.77 (20)	0.725
	No (39)	65.11 (32.92)	67.24 (28.88)	2.14 (23.42)	
Unstable or poor housing conditions	Yes (126)	73.17 (30.77)	75.74 (25.3)	2.57 (24.65)	0.1307
	No (66)	87.99 (16.08)	85.7 (16.39)	-2.29 (11.46)	
Difficulty obtaining food	Yes (147)	74.15 (29.47)	76.61 (24.02)	2.46 (22.05)	0.0648
	No (45)	91.69 (12.9)	87.5 (17.47)	-4.19 (17.18)	
Difficulty paying for utility bills	Yes (19)	76.45 (26.78)	82 (16.79)	5.55 (26.85)	0.3145
	No (173)	78.46 (27.67)	78.85 (23.69)	0.39 (20.47)	

Child abuse	Yes (58)	68.53 (33.54)	74.06 (25.64)	5.53 (-1.32)	0.0458*
	No (134)	82.48 (23.38)	81.37 (21.61)	-1.1 (18.4)	
**Socioeconomic variables at the journey**					
Caregiver's sense that minor was safe/protected	Half of the days or less (147)	76.84 (28.57)	77.92 (23.88)	1.08 (22.21)	0.83
	Most of the days (45)	82.92 (23.48)	83.22 (19.97)	0.31 (17.52)	
Sleeping indoors	Half of the days or less (107)	77.94 (26.14)	78 (22.73)	0.05 (22.1)	0.5356
	Most of the days (85)	78.67 (29.32)	80.63 (23.57)	1.97 (20)	
Access to toilets	Half of the days or less (109)	76.65 (28.04)	77.96 (22.47)	1.31 (24.32)	0.7568
	Most of the days (83)	80.39 (26.86)	80.74 (23.9)	0.36 (16.23)	
Enough drinking water	Half of the days or less (105)	76.01 (28.75)	76.84 (23.65)	0.83 (23.94)	0.9568
	Most of the days (87)	80.98 (25.87)	82.97 (22.18)	0.99 (17.37)	
Enough nutritious food	Half of the days or less (139)	77.09 (28.09)	78.08 (23.23)	0.99 (22.45)	0.9281
	Most of the days (53)	81.34 (25.98)	82.02 (22.65)	0.68 (17.53)	
Access to hygiene measures/products	Half of the days or less (144)	77.77 (27.99)	78.28 (23.36)	0.51 (23.05)	0.6559
	Most of the days (48)	79.74 (26.32)	81.82 (22.24)	2.08 (14.2)	
	Half of the days or less (132)	76.98 (27.52)	77.14 (24.59)	0.16 (20.89)	0.4758

Sufficient clothing, appropriate for the weather	Most of the days (60)	81.1 (27.55)	83.62 (18.78)	2.52 (21.83)	
Always received care when in need of health services	Yes (135)	78.34 (26.94)	79.87 (21.13)	-1.62 (31.38)	0.5128
	No (27)	68.89 (34.7)	67.27 (32.36)	1.53 (20.71)	
Time since leaving place of origin (months)	5 or less (72)	81.63 (23.27)	79.47 (23.08)	-2.16 (19.87)	0.202
	More than 5 to 10 (77)	71.82 (32.4)	75.85 (25.91)	4.03 (24.04)	
	More than 10 (43)	84.16 (22.34)	84.58 (16.05)	0.43 (17.01)	

[1]Comparison of the average difference in scores (travel-departure) for different categories of sociodemographic/socioeconomic variables using Student's t-tests or ANOVA.

[2]The participants from Central America were from Honduras, Guatemala, and El Salvador. The participants from South America were from Venezuela, Colombia, Ecuador, and Peru.

*$p < 0.05$

Supplementary Table 4. Comparison of the evolution of family cohesion in migrant minors with different sociodemographic/socioeconomic profiles.

	***N* = 192**	**Family cohesion subscale**			
	**Categories (*n*)**	Average score last month prior to departure (SD)	Average score last month of travel (SD)	Average score difference (travel-departure) (SD)	*p*-value[1]
**General sociodemographic variables**					
Sex	Male (101)	73.56 (24.39)	66.24 (24.85)	-7.33 (22.72)	0.3527

	Female (91)	70.82 (24.33)	66.59 (26.98)	-4.23 (23.28)	
Age (years)	1-4 (51)	75.1 (23.1)	66.57 (28.36)	-8.53 (26.89)	0.019*
	5-11 (102)	73.92 (24.85)	65.88 (25.55)	-8.04 (21.92)	
	12-17 (39)	64.23 (23.41)	67.56 (23.53)	3.33 (17.82)	
Indigenous/Afro-descendant identity of caregiver	Yes (15)	69.33 (28.28)	52 (24.19)	-17.33 (24.12)	0.0437*
	No (177)	72.51 (24.04)	67.63 (25.64)	-4.89 (22.69)	
**Socioeconomic variables at origin**					
Place of origin[2]	Central America/Mexico (148)	73.07 (23.99)	67.09 (25.8)	-5.98 (23.04)	0.8945
	South America (44)	69.55 (25.56)	64.09 (26.02)	-5.45 (23.05)	
Always received care when in need of health services during the year prior to departure	Yes (147)	73.47 (22.78)	67.79 (25.25)	-5.68 (23.07)	0.6694
	No (39)	68.33 (29.34)	60.9 (27.65)	-7.44 (21.7)	
Unstable or poor housing conditions	Yes (126)	69.96 (25.1)	65.2 (26.26)	-4.76 (22.13)	0.362
	No (66)	76.67 (22.33)	68.71 (24.98)	-7.95 (24.57)	
Difficulty obtaining food	Yes (147)	71.29 (25.54)	64.83 (26.52)	-6.46 (24.1)	0.5124
	No (45)	75.44 (19.82)	71.56 (22.88)	-3.89 (18.98)	

Difficulty paying for utility bills	Yes (19)	67.11 (24)	57.63 (21.37)	-9.47 (22.78)	0.4716
	No (173)	72.83 (24.37)	67.37 (26.13)	-5.46 (23.03)	
Child abuse	Yes (58)	71.38 (27.91)	68.19 (28.85)	-3.19 (24.86)	0.2908
	No (134)	72.65 (22.72)	65.63 (24.46)	-7.01 (22,12)	
**Socioeconomic variables at the journey**					
Caregiver's sense that minor was safe/protected	Half of the days or less (147)	72.93 (23.86)	67.28 (26.48)	-5.65 (23.77)	0.8171
	Most of the days (45)	70.11 (25.99)	63.56 (23.56)	-6.56 (20.44)	
Sleeping indoors	Half of the days or less (107)	70.75 (25.07)	63.08 (25.16)	-7.66 (22.25)	0.2232
	Most of the days (85)	74.18 (23.37)	70.59 (26.17)	-3.59 (23.81)	
Access to toilets	Half of the days or less (109)	69.91 (25.02)	62.98 (25.21)	-6.93 (21.55)	0.4623
	Most of the days (83)	75.36 (23.19)	70.9 (26.05)	-4.46 (24.8)	
Enough drinking water	Half of the days or less (105)	68.48 (24.14)	64.1 (24.32)	-4.38 (22.3)	0.3288
	Most of the days (87)	76.84 (23.91)	69.2 (27.4)	-7.64 (23.78)	
Enough nutritious food	Half of the days or less (139)	70.79 (24.25)	65.83 (24.6)	-4.96 (21.52)	0.3834
	Most of the days (53)	76.13 (24.35)	67.92 (28.96)	-8.21 (26.51)	
Access to hygiene measures/products	Half of the days or less (144)	70.17 (25.02)	65.35 (25.7)	-4.83 (23.58)	0.2819

	Most of the days (48)	78.54 (21.16)	69.58 (26.15)	-8.96 (21.01)	
Sufficient clothing, appropriate for the weather	Half of the days or less (132)	69.81 (24.87)	65.3 (25.75)	-4.51 (23.45)	0.2276
	Most of the days (60)	77.67 (22.37)	68.83 (26.01)	-8.83 (21.81)	
Always received care when in need of health services	Yes (135)	67.78 (29.62)	64.44 (26.47)	-3.33 (14.01)	0.4005
	No (27)	74.26 (22.81)	66.67 (26.28)	-7.59 (25.45)	
Time since leaving place of origin (months)	5 or less (72)	73.33 (24.09)	63.47 (26.08)	-9.86 (24.16)	0.105
	More than 5 to 10 (77)	70.58 (26.18)	68.7 (27.13)	-1.88 (21.76)	
	More than 10 (43)	73.49 (21.51)	67.21 (22.89)	-6.28 (22.39)	

[1]Comparison of the average difference in scores (travel-departure) for different categories of sociodemographic/socioeconomic variables using Student's t-tests or ANOVA.

[2]The participants from Central America were from Honduras, Guatemala, and El Salvador. The participants from South America were from Venezuela, Colombia, Ecuador, and Peru.

*$p < 0.05$
